# Principal-component-based population structure adjustment in the North American Rheumatoid Arthritis Consortium data: impact of single-nucleotide polymorphism set and analysis method

**DOI:** 10.1186/1753-6561-3-s7-s108

**Published:** 2009-12-15

**Authors:** Gina M Peloso, Nadia Timofeev, Kathryn L Lunetta

**Affiliations:** 1 Department of Biostatistics, Boston University School of Public Heath, Crosstown Center, 801 Massachusetts Avenue, 3rd Floor, Boston, Massachusetts 02118 USA

## Abstract

Population structure occurs when a sample is composed of individuals with different ancestries and can result in excess type I error in genome-wide association studies. Genome-wide principal-component analysis (PCA) has become a popular method for identifying and adjusting for subtle population structure in association studies. Using the Genetic Analysis Workshop 16 (GAW16) NARAC data, we explore two unresolved issues concerning the use of genome-wide PCA to account for population structure in genetic associations studies: the choice of single-nucleotide polymorphism (SNP) subset and the choice of adjustment model. We computed PCs for subsets of genome-wide SNPs with varying levels of LD. The first two PCs were similar for all subsets and the first three PCs were associated with case status for all subsets. When the PCs associated with case status were included as covariates in an association model, the reduction in genomic inflation factor was similar for all SNP sets. Several models have been proposed to account for structure using PCs, but it is not yet clear whether the different methods will result in substantively different results for association studies with individuals of European descent. We compared genome-wide association *p*-values and results for two positive-control SNPs previously associated with rheumatoid arthritis using four PC adjustment methods as well as no adjustment and genomic control. We found that in this sample, adjusting for the continuous PCs or adjusting for discrete clusters identified using the PCs adequately accounts for the case-control population structure, but that a recently proposed randomization test performs poorly.

## Background

Cases for the North American Rheumatoid Arthritis Consortium (NARAC) study were collected across the United States; controls were collected from Long Island, NY [1]. Because of the differences in the mix of European ancestry across the US, we expect to see population structure in the Genetic Analysis Workshop 16 (GAW16) NARAC sample. Population structure can lead to spurious association when the distributions of both the trait and the genotype of interest vary among subpopulations. Even modest differences among subpopulations can lead to spurious associations in large samples: population structure bias is of particular concern for genome-wide studies in which large numbers of subjects are needed to detect modest effects of a single-nucleotide polymorphism (SNP) with the phenotype [2].

Several methods have been developed to detect and adjust for population structure, including genomic control [3], structured association [4], and genome-wide principal-components analysis (PCA) [5,6]. Here, we focus on the PCA approach. The principal components (PCs) are computed using the genotype matrix from genome-wide SNP data, where each SNP genotype is a count (0, 1, or 2) of the number of copies of the minor allele [5,6]. Each PC is a weighted sum of all SNP genotype counts. The first PC accounts for the largest proportion of genetic variation; each additional PC accounts for successively smaller proportions. The PCs have been shown to capture genetic differences among individuals of European heritage due to ancestry [7]. These components can be used as covariates in an association analysis to account for differences in ancestry among individuals [6].

When limited numbers of SNPs are available to compute the PCs, each SNP will contribute a greater proportion of information to the PCA. In this situation, SNPs in strong linkage disequilibrium (LD) may have excessive influence on one or a set of PCs [5]. However, it is not clear whether the same issue arises in the context of dense genome-wide SNPs, where some investigators have advocated for the use of all SNPs to compute the PCs [5], while others have proposed the use of a subset of SNPs in linkage equilibrium [8]. In previous analyses of the NARAC data set, investigators noted that the third and fourth PCs from a genome-wide PCA heavily weighted SNPs within a 3.8-Mb region on 8p23 with a known inversion polymorphism [1], and advocated the omission of SNPs in this region, as well as the MHC region that is strongly associated with RA. Thus, conflicting views remain on how best to select a set of SNPs to use for PCA for the purpose of adjustment for population structure in genetic association studies.

In addition to the controversy concerning choice of SNPs for PCA, investigators have proposed several methods to adjust for the structure. The developers of the computer program EIGENSTRAT recommend adjustment for the PCs as linear covariates in an association model, while other investigators have proposed approaches that cluster individuals, arguing that the PCs, phenotype, and allele frequencies may not be linearly related, particularly when the PCs create distinct clusters [9]. Kimmel et al. propose a randomization test as an alternative to adjusting for PCs within a traditional regression framework [10]. Using simulation, they show that their population structure association test (PSAT) is considerably more powerful than the linear adjustment approach of EIGENSTRAT in some situations [10]. It is unclear whether the proposed approaches will result in substantively different results in typical genome-wide association studies using individuals of European ancestry.

To address these questions, we compare six choices for subsetting SNPs for PCA, and four methods for adjusting for structure identified using PCA, as well as no adjustment and genomic control, using the NARAC rheumatoid arthritis (RA) genome-wide SNP data provided by GAW16. Our goal is to determine to what extent SNP choice and method of population structure adjustment affects the results of the genome-wide association study, measured by the genomic control inflation factor, and by specific association results for previously reported positive associations between SNPs and RA [11-13].

## Methods

### Quality control

GAW16 NARAC samples (868 cases, 1194 controls) were genotyped using the Illumina 550 k chip. All subjects had fewer than 10% missing genotypes. Seven subjects had X chromosome heterozygosity incompatible with their phenotypic sex (homozygosity > 0.2 for females or <0.8 for males) [14] and were omitted from all analyses. We excluded SNPs with departure from Hardy-Weinberg equilibrium in controls (*p *< 10^-6^), ≥ 2% missing genotypes, or minor allele frequency <0.01, leaving a total of 470,943 autosomal SNPs.

### SNP subsets for PCA

We computed the genotypic PCs using the 2055 NARAC individuals for six subsets of SNPs: (S1) all SNPs passing quality control ("filtered SNPs"), (S2) filtered SNPs, omitting SNPs in the MHC region, (S3) filtered SNPs omitting SNPs in the MHC region and the inversion polymorphism on chromosome 8, (S4) filtered SNPs omitting all of chromosome 6 and the inversion polymorphism on chromosome 8, (S5) filtered SNPs omitting one SNP from each pair of SNPs having allelic correlation *r*^2 ^> 0.4 [14], and (S6) filtered SNPs omitting one SNP from each pair of SNPs with *r*^2 ^> 0.2.

For SNP Sets (S2) and (S3), we omitted all SNPs within 10 kb of 6q21.3 [15], the MHC region that is highly associated with RA. We defined the chromosome 8 inversion polymorphism as the region spanning 8 Mb to 11.5 Mb [1]. Previous studies have omitted these regions when conducting genome-wide PCA to avoid bias due to a region strongly linked to case-status, and a region with known high LD and extreme PC SNP weights, respectively [1].

### Adjustment for structure in association analyses

To simplify presentation, we selected a single SNP subset (S3, filtered SNPs omitting MHC and inversion polymorphism SNPs) for exploration of adjustment methods (see Results for rationale for the choice of Set S3). We determined the distributions of genome-wide *p*-values excluding the highly associated MHC region and association results for two specific SNPs that have been reported to be associated with RA: rs2476601 [11,12] and rs3761847 [1] for six models: (A) logistic regression adjusting for the DRB locus (no adjustment for structure), (B) logistic regression adjusting for the DRB locus and PC1-4 as continuous covariates, (C) logistic regression adjusting for the DRB locus and the five clusters obtained by clustering, (D) logistic regression adjusting for the DRB locus, PC1-4 as continuous covariates, and the five PC clusters, (E) PSAT [10] with disease probability assigned based on the five PC-based clusters, and (F) genomic control using the logistic model adjusting for the DRB locus. For all analyses, we coded the SNP genotype as the count of the number of minor alleles. This model assumes a linear relationship between the log odds of case status and the number of minor SNP alleles. The first four PCs for SNP Subset (S3) were associated with RA status (Wilcoxon rank sum test *p*-value < 0.05); we therefore used these PCs for adjustment and clustering in association analyses [Methods (B)-(E)]. For association Models (C), (D), and (E) we used the partitioning around medoids algorithm ("pam" function in R [16]) to cluster the individuals using the four PCs associated with RA case status. A five-cluster solution was optimal based on silhouette widths. The DRB locus is defined as the number of high- and medium-risk alleles each individual carried [15].

Logistic regression analyses [Models (A)-(D)] were performed using the computer program PLINK [14] and analysis for Method (E) was performed using the PSAT software [10]. PSAT tests the association between a SNP and disease, accounting for population structure using a randomization test. The user specifies the probability of being a case for each cluster based on ancestry information. For each replicate, case status for each individual is re-assigned at random according to the baseline probability for the cluster to which the individual is assigned. The probabilities of disease and sample sizes in the five clusters were 0.08 (*n *= 77), 0.11 (*n *= 68), 0.12 (*n *= 460), 0.37 (*n *= 230), and 0.63 (1120). One million samplings were used to assess significance.

## Results

### SNP subsets

**Figure 1:**
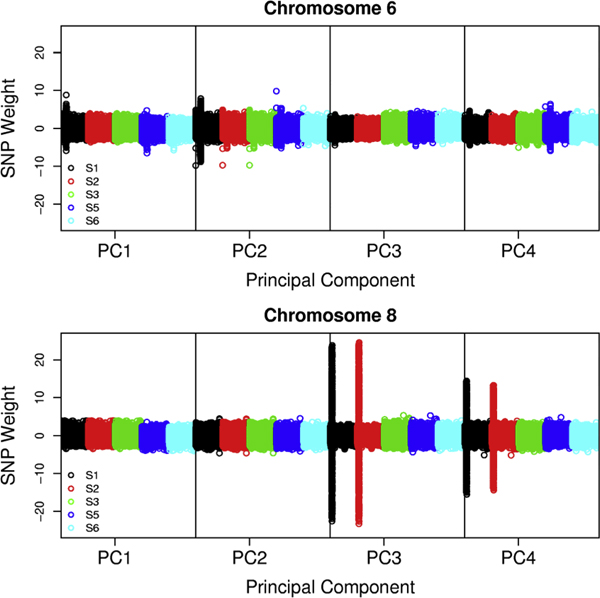
**PCA plot of SNP weights**. Plot of SNP weights from PCAs S1 (black), S2 (red), S3 (green), S5 (blue), and S6 (turquoise).

For each SNP subset, PC1-3 are highly associated with case status (two-sample Wilcoxon test *p *< 0.05; Table 1). Association of other PCs with case status varied depending on the subset. PC1 and PC2 for the S3 subset, which excluded the MHC region and the inversion polymorphism on chromosome 8, are highly correlated with the corresponding PC in all other subsets (square of the Pearson correlation coefficient *r*^2 ^> 0.94). For each of the remaining S3 PCs, we display in Table 2 the PCs from the other SNP subsets that are correlated with *r*^2 ^> 0.15. Subset S3 PC3 and PC4 were highly correlated with the same PCs for subsets S4-S6 (*r*^2^: 0.83-0.98). For Subset S2, PC3 and PC4 appear to take the place of S3 PC3. The correlations between the S3 PCs and PC3-PC10 obtained from the minimally filtered SNP Set S1 are weaker and less consistent. We examined the SNP weights to understand why some PCs in different subsets are not strongly correlated. SNPs in the MHC region on chromosome 6 were assigned large weights for PCs 1 and 2 in the S1 subset. Removal of SNPs in this region (S2, S3, and S4), or general removal of SNPs in LD across the genome (S5, S6) reduced the problem of extreme weights on chromosome 6 (Figure 1). SNPs in the inversion region on chromosome 8 were assigned extreme weights for PC3 and PC4 for SNP Subsets S1 and S2 (Figure 1). SNP subsets omitting this region (S3, S4) or omitting SNPs in LD (S5, S6) showed no evidence for overweighting on chromosome 8. The four PCs associated with RA status in the subsets that removed the MHC region and the chromosome 8 inversion, (S3 and S4), or filtered out SNPs in high LD (S5 and S6) exhibited minimal extreme SNP weights across the genome other than a small region on chromosome 2 that contains the lactase gene (*LCT*), which is known for strong structure in European populations [17].

**Table 1:** Summary of PCA with six SNP subsets

SNP Subset for PCA	**No**. SNPs	PCs significantly associated with RA (α = 0.05)	λ^a^
S1. Filtered SNPs	459,422	1, 2, 3, 5, 6, 8, 9	1.023
S2. Removing the MHC region	457,776	1, 2, 3, 5	1.027
S3. Removing MHC and inversion on chr 8	456,846	1, 2, 3, 4	1.028
S4. Removing chr 6 and inversion on chr 8	427,806	1, 2, 3, 4	1.030
S5. Removing LD between SNPs with *r*^2 ^> 0.4	164,418	1, 2, 3, 4	1.022
S6. Removing LD between SNPs with *r*^2 ^> 0.2	81,240	1, 2, 3, 4, 5	1.023

^a^Inflation factor for genome-wide SNP association test statistics adjusting for PCs significantly associated with RA at (α = 0.05) as linear covariates

**Table 2:** Correlation between S3 PCs and other SNP subset PCs.

PCA removing MHC and inversion on chromosome 8 (S3)^b^								
**Subset**	**PC3**	**PC4**	**PC5**	**PC6**	**PC7**	**PC8**	**PC9**	**PC10**

S1	PC3, PC4 (0.32,0.66)	PC5, PC6 (0.55, 0.81)	PC5, PC6 (0.46, 0.27)	PC7 (0.92)	PC8, PC9, PC10 (0.35, 0.25, 0.37)	PC9 (0.20)	----	----
S2	PC3, PC4 (0.29, 0.72)	PC5 (0.98)	PC6 (0.98)	PC7 (0.98)	PC8 (0.99)	PC9 (0.98)	PC10 (0.96)	----
S4	PC3 (0.98)	PC4 (0.98)	----^c^	PC5 (0.92)	PC6 (0.85)	PC7, PC8 (0.61, 0.18)	PC7, PC8 (0.16, 0.58)	PC9 (0.66)
S5	PC3 (0.96)	PC4 (0.90)	----	----	----	----	----	----
S6	PC3 (0.92)	PC4 (0.83)	----	----	----	----	----	----

^a^For each SNP subset, the PCs correlated with the corresponding subset S3 PC with *r*^2 ^> 0.15 are displayed, along with the *r*^2 ^value.

^b^PC1 and PC2 for each subset are correlated with S3 subset PC1 and PC2 (*r*^2 ^> 0.98, *r*^2 ^> 0.94), and are not shown.

^c^---, No PC has correlation (*r*^2 ^> 0.15) with the S3 subset PC.

### Adjustment for structure

When using PCA to account for population structure, the primary goal is to reduce type I error to the nominal (expected) level without reducing power for true positive associations. The genomic control inflation factor (λ), defined as the median association test statistic across all SNPs divided by its expected value, is an overall indicator of the inflation in the test statistics [4]. We computed λ for genome-wide analyses excluding the MHC region, adjusting for the PCs significantly associated with RA at α = 0.05 as linear covariates. For the six SNP subsets, λ ranged between 1.022 and 1.030, indicating good control of inflation compared to the substantial inflation (λ = 1.24) observed for analyses unadjusted for structure (Table 1).

Linear adjustment for PCs to account for population structure has been shown to have lower power and higher type I error than alternatives in some situations [9,10], therefore we explored other methods for adjusting for structure. To simplify presentation, we selected a single SNP subset (S3) for exploration of adjustment methods. SNP Sets S1 and S2 produced several PCs that were significantly associated with RA and also had heavily weighted SNPs in some genomic regions (Figure 1). Because heavy weights in a region will reduce the power to detect true associations with SNPs in that region, we selected the S3 SNP subset, which used the maximum number of SNPs to determine the PCs while minimizing overweighting of SNPs in any region.

**Figure 2:**
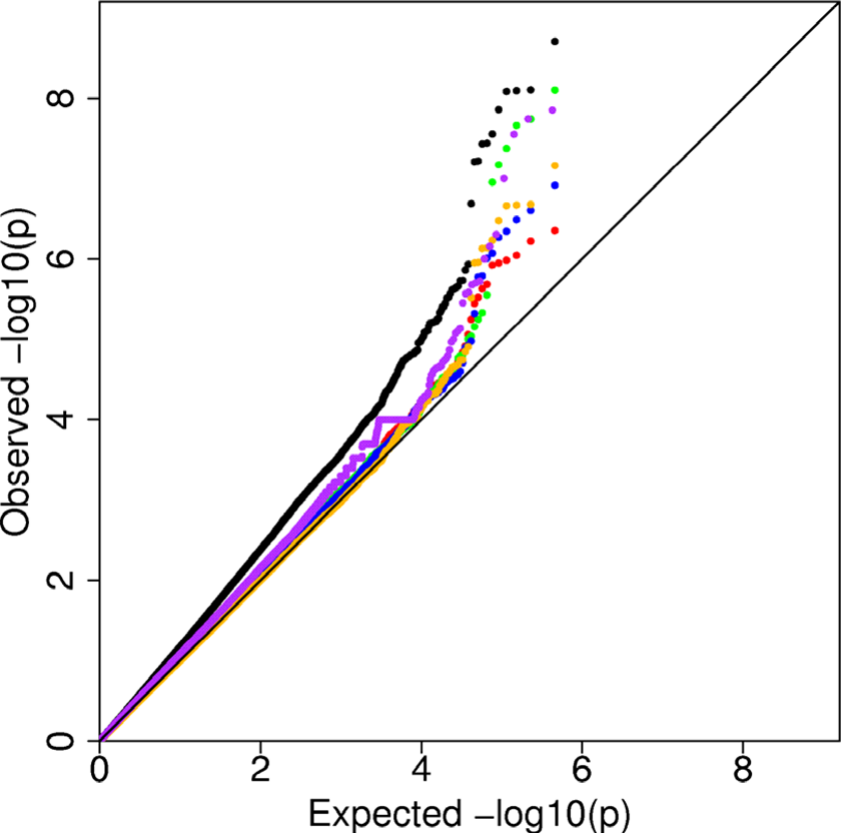
**Q-Q plot of -log_10_(*p*-value) for analyses**. Quantile-Quantile plot of -log_10_(*p*-value) for Methods A-F as described in Methods. For each analysis, we plot the negative -log_10_(*p*-value) for all genome-wide SNPs, excluding the SNPs in the MHC. Black, Method (A) - logistic regression adjusting for the DRB locus (inflation factor λ = 1.24); Red, Method (B) - logistic regression adjusting for the DRB locus and PC1-4 as continuous covariates (λ = 1.03); Green, Method (C) - logistic regression adjusting for the DRB locus and PC cluster (λ = 1.04); Blue, Method (D) - logistic regression adjusting for the DRB locus, PC1-4 as continuous covariates, and PC cluster (λ = 1.03); Purple, Method (E) - PSAT with disease probability assigned based on clustering on PC1-4 (λ = 1.15); and Orange, Method (F) - genomic control using the logistic model adjusting for the DRB locus (λ = 1.00).

For the genome-wide results excluding the MHC region, we compared the genomic control inflation factor (λ) for adjustment Methods (B)-(E) to the unadjusted Model (A). The inflation factor was 1.24 for the unadjusted logistic regression, 1.03-1.04 for the PC-adjusted logistic regression analyses, and 1.15 for the PSAT analysis (Figure 2). These findings suggest that adjusting either for PC1-PC4 or the cluster assignments based on PC1-PC4 considerably reduces the inflation in the test statistic, but that adjusting for both PC1-PC4 and cluster membership does not further reduce the inflation for these data. The PSAT randomization test also reduces the inflation, but not nearly as well as the less computer intensive approaches for adjusting for structure. The association results for two SNPs previously found to be associated with RA are displayed in Table 3. Both SNPs have differences of more than 0.15 in allele frequencies across HapMap populations, and are therefore also likely to have allele frequency differences across European populations. All adjustment methods reduce the significance of the SNPs but still provide evidence for association.

**Table 3:** Results for positive control SNPs for six methods of adjusting for structure

	**rs2476601 **[11,12]		**rs3761847 **[1]	
		
Adjustment Method	β^a ^± SE	*p*-Value	β^a ^± SE	*p*-Value
A. Logistic regression adjusting for DRB	0.61 ± 0.11	3.70 × 10^-8^	0.42 ± 0.07	8.04 × 10^-9^
B. Logistic regression adjusting for DRB and PC1-4	0.47 ± 0.12	1.32 × 10^-4^	0.39 ± 0.08	2.08 × 10^-6^
C. Logistic regression adjusting for DRB and cluster	0.46 ± 0.12	1.57 × 10^-5^	0.45 ± 0.08	4.21 × 10^-8^
D. Logistic regression adjusting for DRB, PC1-4, and cluster	0.46 ± 0.13	2.44 × 10^-4^	0.42 ± 0.08	4.55 × 10^-7^
E. PSAT based on PC1-4 cluster	-----^b^	1.17 × 10^-6^	-----	2.80 × 10^-8^
F. Genomic control	0.16 ± 0.12	7.44 × 10^-7^	0.42 ± 0.08	2.22 × 10^-7^
Previously reported	0.50 ± 0.15	6.60 × 10^-4^	0.35 ± 0.04	4.00 × 10^-14^

^a^β, log-odds ratio for disease for the minor allele estimated by the logistic regression

^b^---, Estimates of effect cannot be obtained.

## Conclusion

We performed PCA for six subsets of genome-wide SNPs; the first two PCs were similar for all subsets, and were highly associated with RA case status. The third and fourth PCs were very similar for SNP sets that either removed all SNPs in LD or removed the chromosome 8 inversion region known for high LD. SNP sets that included all SNPs had SNPs with extreme weights for some PCs. In this data set, structure adjustment using PCs obtained from any of the SNP subsets similarly reduces the genomic inflation factor. However, SNP sets that result in PCs that over-weight a genomic region may be prone to loss of power because strong PC weights on a true-positive SNP may adjust away the SNP effect.

We evaluated four methods of adjusting for structure using PCs. Adjustment for the four PCs associated with case status as linear covariates or for the cluster assignments in logistic regression substantially reduces both the genomic inflation factor and the significance level of the most highly associated SNPs (Figure 2), suggesting that the structure that exists in this sample does result in some bias. A criticism of adjusting for PCs as linear covariates is that the PCs and disease frequency may not be linearly related, especially when the PCs create distinct clusters [9]. However, in this sample we found that adjusting for either cluster assignment or PCs as linear covariates in logistic regression produced similar genomic inflation factor (λ) and comparable effect estimates for two specific positive control SNPs. Although the PSAT randomization method has been reported to provide improved power and better control of type I error [9,10], in this sample the method produced a considerably higher inflation factor for the genome-wide test statistics than the other structure adjustment methods (Figure 2). Because PSAT relies on clustering individuals based on PCs or other distance metrics, it may ultimately be more useful for accounting for population structure at the cross-continent level, rather than the more subtle structure observed in populations of European descent.

Exploring existing genome-wide association study data allows us to gain experience with methods for adjusting for population structure under realistic conditions, including variable LD due to inversion polymorphisms and regions of highly associated SNPs. We have found that population structure exists in the GAW16 RA data, and that the structure is associated with case status. Therefore, the association tests are potentially subject to confounding by genetic ancestry. In this sample, we have determined that the choice of SNP subset for PCA does not substantively affect the resulting reduction in genomic inflation factor for genome-wide association analyses. However, for some SNP subsets, the PCs associated with case status and used in the structure adjustment heavily weighted small numbers of SNPs, which could lead to reduced power to detect true associations in those genomic regions. Thus, the SNP set selected could have an impact on power. We recommend that in practice, for studies with modest levels of structure such as that expected when subjects are all of European ancestry, investigators check the PCs that will be used for structure adjustment for extreme weights and choose alternative SNP sets if any genomic regions are heavily weighted.

## List of abbreviations used

GAW16: Genetic Analysis Workshop 16; LD: Linkage disequilibrium; NARAC: North American Rheumatoid Arthritis Consortium; PC: Principal component; PCA: Principal-component analysis; PSAT: Population structure association test; RA: Rheumatoid arthritis; SNP: Single-nucleotide polymorphism.

## Competing interests

The authors declare that they have no competing interests.

## Authors' contributions

GMP and NT participated in carrying out the analysis, interpreting the data, and drafting the manuscript. KLL participated in interpreting the data and drafting and critically revising the manuscript. All authors read and approved the final manuscript.
